# In This Issue

**Published:** 1995

## PAST ACCOMPLISHMENTS AND FUTURE GOALS

Dr. Enoch Gordis, director of the National Institute on Alcohol Abuse and Alcoholism (NIAAA), reviews the significant developments in alcoholism research of the past 25 years. Thanks largely to NIAAA support, alcoholism researchers have made significant strides in understanding the causes, prevention, and treatment of alcoholism and its consequences. Ongoing research will extend this knowledge. (pp. 5–11)

## THE CREATION OF NIAAA

Late in 1970 the legislation creating NIAAA was signed into law. It was the first law since Prohibition to provide a coordinated effort to address the problems of alcohol abuse and alcoholism nationwide. Ms. Brenda G. Hewitt recounts the extraordinary efforts made by a handful of dedicated citizens and legislators to see this law passed. She also reviews the continually shifting attitudes toward alcohol consumption and alcohol problems that have characterized the history of attempts by Americans to define this substance’s place in society. (pp. 12–16)

## NIAAA’S DIRECTORS LOOK BACK ON 25 YEARS

NIAAA has been guided by seven directors over the course of its 25-year history. This series of interviews with the six living directors chronicles their involvement in the field of alcohol research and policy-making and presents their views on the past, present, and future of NIAAA. (pp. 17–27)

## SEMINAL ARTICLES IN ALCOHOL RESEARCH

This special section features commentaries from experts in the alcohol field on original articles that have described some of the most important alcohol research of the past four decades. These landmark articles include studies describing alcohol’s affects on the body, such as alcohol-withdrawal syndrome and fetal alcohol syndrome as well as alcohol’s impact on the heart and liver; the pioneering use of animal models in alcohol research; different types of alcoholism treatment, including pharmacological and clinical trials; the role that genetics may play in the inheritance of alcohol problems; and the effect of legislative policy changes and economic forces on drinking behavior. (pp. 28–59)

## NIAAA’S INTRAMURAL RESEARCH PROGRAM

Since its inception as an independent Institute within the National Institute of Mental Health, NIAAA’s Intramural Research Program (IRP) has maintained a strong research tradition. Drs. Markku Linnoila, Theodore R. Colburn, and Robert C. Petersen detail ongoing studies in the IRP. They also review the program’s training and collaborative efforts with scientists and other researchers within the National Institutes of Health and enumerate the IRP’s future research directions. (pp. 60–70)

## ALCOHOL HEALTH SERVICES RESEARCH

Alcohol health services research primarily is concerned with analyzing factors that affect the delivery of alcoholism services to clients in treatment settings. Dr. Constance Weisner details the general scope of health services research, describes influences that have affected the delivery of treatment services over the past two decades and that have had implications for alcohol-related health services research, and poses important questions for the future of this branch of alcohol research. (pp. 71–76)

## TWENTY-FIVE YEARS OF ALCOHOL EPIDEMIOLOGY

Alcoholism has become a topic of epidemiological research only since the beginning of the 1970’s. Dr. Mary C. Dufour reviews the development and features of alcohol epidemiology and describes NIAAA’s involvement in efforts to determine patterns and consequences of alcohol consumption and abuse in the United States. (pp. 77–84)

